# Increased risk of death in COVID-19 hospital admissions during the second wave as compared to the first epidemic wave: a prospective, single-centre cohort study in London, UK

**DOI:** 10.1007/s15010-021-01719-1

**Published:** 2021-10-21

**Authors:** Martina Cusinato, Jessica Gates, Danyal Jajbhay, Timothy Planche, Yee Ean Ong

**Affiliations:** 1 Institute for Infection and Immunity, St. George’s University of London, London, UK; 2 Department of Respiratory Medicine, St. George’s University Hospitals NHS Foundation Trust, London, UK; 3 Institute of Medical and Biomedical Education, St. George’s University of London, London, UK

**Keywords:** COVID-19, Hospital mortality, SARS-CoV-2, SARS-CoV-2 variants, Epidemiology

## Abstract

**Background:**

The second coronavirus disease (COVID-19) epidemic wave in the UK progressed aggressively and was characterised by the emergence and circulation of variant of concern alpha (VOC 202012/01). The impact of this variant on in-hospital COVID-19-specific mortality has not been widely studied. We aimed to compare mortality, clinical characteristics, and management of COVID-19 patients across epidemic waves to better understand the progression of the epidemic at a hospital level and support resource planning.

**Methods:**

We conducted an analytical, dynamic cohort study in a large hospital in South London. We included all adults (≥ 18 years) with confirmed severe acute respiratory syndrome coronavirus 2 (SARS-CoV-2) who required hospital admission to COVID-19-specific wards between January 2020 and March 2021 (*n* = 2701). Outcome was COVID-19-specific in-hospital mortality ascertained through Medical Certificate Cause of Death.

**Results:**

In the second wave, the number of COVID-19 admissions doubled, and the crude mortality rate dropped 25% (1.66 versus 2.23 per 100 person-days in second and first wave, respectively). After accounting for age, sex, dexamethasone, oxygen requirements, symptoms at admission and Charlson Comorbidity Index, mortality hazard ratio associated with COVID-19 admissions was 1.62 (95% CI 1.26, 2.08) times higher in the second wave.

**Conclusions:**

Although crude mortality rates dropped during the second wave, the multivariable analysis suggests a higher underlying risk of death for COVID-19 admissions in the second wave. These findings are ecologically correlated with an increased circulation of SARS-CoV-2 variant of concern 202012/1 (alpha). Availability of improved management, particularly dexamethasone, was important in reducing risk of death.

**Supplementary Information:**

The online version contains supplementary material available at 10.1007/s15010-021-01719-1.

## Introduction

Since its emergence in December 2019, the spread of severe acute respiratory syndrome coronavirus 2 (SARS-CoV-2) has posed immense challenges for health care systems across the globe [1]. In the United Kingdom (UK) the first confirmed case was registered on 31 January 2020, but transmission rates increased quickly leading to the introduction of a series of control measures that escalated to a full national lockdown (23 March 2020). This was subsequently followed by a drop in transmission and hospitalisation rates with restrictions eased over the summer months. However, in October 2020 infections began to increase again leading to a second wave of coronavirus disease (COVID-19) cases. The implementation of a second lockdown (5 November 2020) followed by tiered control measures were needed to reduce the transmission rates again [2]. As of 1 May 2021, the UK has recorded 4,418,819 confirmed cases, 463,485 hospital admissions, and 127,571 deaths [3].

During the first wave of COVID-19, relatively little was known about this novel illness and patient management was largely based upon experience of treating other viral infections. However, as new evidence became available, the standard of care of patients admitted with COVID-19 changed. From the start of the second wave, dexamethasone was prescribed to all patients requiring supplemental oxygen and Remdesivir was administered to hypoxemic patients presenting within 10 days of symptoms onset. The indications for Tocilizumab changed during the second wave where patients initially had access to this only within clinical trials [4–8].

A key feature of the second wave of COVID-19 in the UK, was the emergence of a new SARS-CoV-2 variant designated VOC 202012/01 or alpha (lineage B.1.1.7). This new variant was identified in samples originally taken in South East England in early October 2020, and became the predominant variant circulating in the UK throughout the second epidemic wave [9–11]. It is now established that VOC 202012/01 is more transmissible than pre-existing variants and it is associated with an excess of all-cause mortality 28 days after a positive test in the community [12–14]. However, the impact of this VOC on in-hospital COVID-19-specific mortality remains poorly understood.

St George’s University Hospital NHS Foundation Trust is one of the largest hospitals in the UK and is based in South West London. It serves a local catchment population of 560,000 and specialist services to 3.4 million people. The objective of this study was to assess whether mortality of patients admitted for COVID-19 treatment was different in the second UK wave of COVID-19 compared to the first wave accounting for differences in the standard of care available in each wave.

## Methods

### Study design

This is a single-centre, analytical, dynamic cohort study using data extracted from routinely collected, electronic medical records and hospital database.

### Participants and setting

The study population for this cohort study comprised all adults (≥ 18 years) with SARS-CoV-2 infection confirmed by polymerase chain reaction (PCR) and/or clinico-radiological diagnosis of COVID-19, who required hospital admission to COVID-19-specific wards at St George’s University Hospitals NHS Foundation Trust (London, UK). Patients seen in the Emergency Department or in Acute Medical Units (AMU) who were discharged on the same day were not included. Although COVID-19 wards opened in March 2020, the study period encompasses admissions between 1 January 2020 and 31 March 2021, as some of the early patients admitted to COVID-19 wards were already hospitalised. All patients meeting the inclusion criteria during the study period were included in the cohort. There was no a priori study size calculation.

### Data sources and measurement

The study cohort was identified retrospectively using hospital records of admissions to active COVID-19 wards. These lists included patient identifiers, hospital admission date, ward and administrative information. Respiratory and intensive care clinicians within the study team and involved in the care of COVID-19 patients, reviewed the electronic medical records for all the patients in the initial list, confirming criteria for COVID-19 admission. In case of patients with multiple COVID-19 admissions, only the most severe, as defined by the highest respiratory support needed, was included [15].

Study follow-up was also carried out by clinicians, prospectively, through review of electronic medical records. Patient data were extracted manually using a standardised electronic questionnaire and was supervised by a senior clinician within the respiratory team. Some data were also obtained through the informatic department and linked using hospital identifiers (laboratory, pathology results and ethnicity data).

The follow-up period for this study began at admission and ended at outcome occurrence (death) or censoring. Participants were censored at hospital discharge or at 6 months if admissions exceeded this period (one patient only).

PCR pathology results were available for all tests requested during the study period, so we matched these with our cohort of patients. Those with positive PCR results dated at least 15 days after their hospital admission were considered probable hospital acquired infections (HAI) and had the start of their follow-up (time at risk) amended to be 14 days (maximum incubation period [16]) before the date of the positive PCR result, instead of the actual admission day.

### Variables

**Fig. 1:**
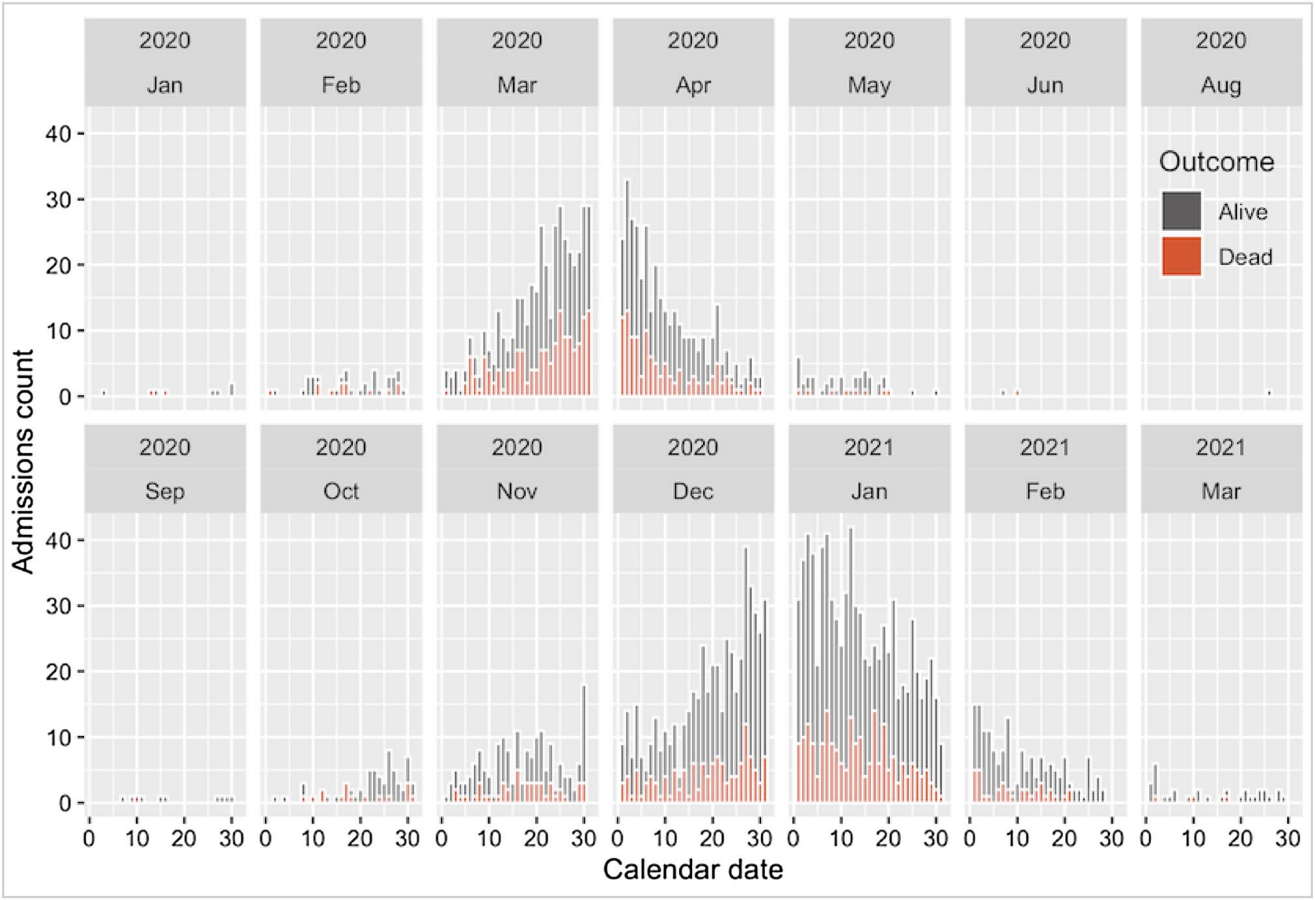
Number of admissions per day according to outcome

The outcome variable was in-hospital COVID-19-associated mortality (as cause or contributor), ascertained from clinical records and Medical Certificate Cause of Death (MCCD). The main explanatory variable for this analysis was COVID-19 wave, and 31 June 2020 used as cutoff to separate both waves. UK Office for National Statistics estimated the end of the first wave at the end of May 2020, and the beginning of the second wave at the beginning of September 2020 [17]. There were three patients admitted between these dates (Fig. 1), two admissions at the beginning June 2020 were considered first wave, and one at the end of August 2020 was considered second wave. First wave was used as baseline/reference.

Covariates of interest for this analysis included demographics (sex, age at admission, ethnicity), symptoms at admission, body mass index (BMI), treatment (dexamethasone, Remdesivir, Tocilizumab), oxygen requirement, HFNO/CPAP (high flow nasal oxygen/continuous positive airway pressure administered only in specialist wards), invasive ventilation, COVID-19 pneumonitis admitted to Intensive Care Unit (ICU) admission, Clinical Frailty Score (CFS), Charlson Comorbidity Index (CCI). Most variables were used in their original scale, others were recategorised using clinically relevant categories with a sufficient number of participants in each group to avoid sparsity.

Where categorised, age groups in years were: 18–39, 40–59, 60–79, ≥ 80. BMI at admission was grouped using categories derived from the WHO classification of BMI (in kg/m^2^): < 18.5, 18.5–24.9, 25–29.9,  ≥ 30 [18]. Oxygen requirement was a dichotomous variable indicating whether the maximum FiO2 (Fraction of Inspired Oxygen) was over 21%. Symptoms at admission were respiratory or wider infective symptoms at time of presentation. The CFS level was collected on a nine-point ordinal scale to assess frailty within 2 weeks of admission, but to avoid sparsity categories seven to nine were grouped [19]. CFS was expanded to include all age groups excepting those patients with disabilities which rendered it inappropriate. CCI is a widely used comorbidity summary measure, based on age and a predefined number of conditions with an assigned integer weight representing the severity of each condition; for this analysis, scores of eight or more were grouped in one category [20]. Individual comorbidities were not included to avoid collinearity with CCI. All scores were calculated by clinicians experienced in the use of scales.

### Statistical methods

The distribution of covariates was assessed for the entire cohort and across waves. Chi-square test was used to assess independence. Mortality rates and person-time of observation were calculated for the main exposure groups and all covariates of interest. The strength of the association was quantified using incidence rate ratios (IRR), and the statistical significance using 95%CIs and p values. Survival across the different waves was explored using time-to-event analysis and log-rank to test the significance of the difference between the survival curves.

Rates were modelled using Cox regression for the multivariable analysis. Proportional hazard assumption was supported (graphically and by testing for a zero slope in Schoenfeld residuals).

A causal model was built using a stepwise backward approach where (non-forced) pre-defined covariates were retained in the model unless there were problems with multicollinearity. Age and gender were considered a priori confounders (forced variables). Age was fitted using restricted cubic splines, with knots positioned so numbers of events between knots were approximately equally distributed. The full model included age, gender and all variables found to confound the crude association between wave and mortality (non-forced variables). A change in the magnitude ≥ 5.5% was considered an indication of confounding. ICU admission was included in the model as a non-forced variable regardless of the degree of confounding of the main association. Problems with multicollinearity on the main effect in the full model, were resolved using RMSE (Root Mean Square Error) reduction for backward deletion of non-forced variable, with RMSE for the full model used as reference for each step [21].

Following the same methodology, we did a sub-analysis among those requiring ICU admission. Data management and statistical analysis were carried out using R (R Core Team version 3.6.3, Vienna, Austria).

### Governance and ethics

This study was approved by the Health Research Authority (20/SC/0220). This manuscript follows the STROBE statement for reporting of cohort studies.

## Results

### Participants

Between 1 January 2020 and 31 March 2021, there were 3376 COVID-19 positive adult patients registered at St. Georges Hospital. Of these, 2701 were patients admitted to COVID-19 wards for treatment, all of whom were included: 32.7% (884 of 2701) in the first wave and 67.3% (1817 of 2701) in the second wave (Fig. 1). At the time of database lock (23^rd^ May 2021), there were 16 patients (all admitted during the second wave) with no outcome recorded.

The distribution of characteristics at admission for the entire cohort and across waves is shown in Table 1. COVID-19 patients admitted during the second wave were more likely to be younger with intermediate levels of frailty: CFS 3 to 5 were more prevalent in the second wave (1103 of 1817, 60.7%) than in the first wave (392 of 884, 44.3%). Admissions scoring zero to 3 in CCI were more prevalent during the second wave (891 of 1817, 49.0% vs. 386 of 884, 43.7%). Absence of respiratory or wider infective symptoms at onset (i.e., hospital acquired infections with initial diagnosis through PCR) was more prevalent in the second wave (361, 19.9%) compared to the first wave (50, 5.7%).

**Table 1:** Baseline characteristic of the study population and comparison groups

Variable	Categories	First wave	Second wave	Total	*p *value
		*N* = 884 (100.0%)	*N *= 1817 (100.0%)	*N *= 2701 (100.0%)	
Sex	Female	363 (41.1%)	782 (43.0%)	1145 (42.4%)	0.330
	Male	521 (58.9%)	1035 (57.0%)	1556 (57.6%)	
Age	18–39	71 (8.0%)	147 (8.1%)	218 (8.1%)	0.001
Grouped	40–59	190 (21.5%)	495 (27.2%)	685 (25.4%)	
(years)	60–79	350 (39.6%)	724 (39.8%)	1074 (39.8%)	
	≥ 80	273 (30.9%)	451 (24.8%)	724 (26.8%)	
Ethnicity	White	301 (34.0%)	636 (35.0%)	937 (34.7%)	< 0.001
	Asian	145 (16.4%)	346 (19.0%)	491 (18.2%)	
	Black	143 (16.2%)	252 (13.9%)	395 (14.6%)	
	Other	147 (16.6%)	491 (27.0%)	638 (23.6%)	
	Unknown	148 (16.7%)	92 (5.1%)	240 (8.9%)	
Clinical	1: Very fit	94 (10.6%)	102 (5.6%)	196 (7.3%)	< 0.001
Frailty	2: Fit	119 (13.5%)	173 (9.5%)	292 (10.8%)	
Score (CFS)	3: Managing well	217 (24.5%)	685 (37.7%)	902 (33.4%)	
	4: Vulnerable	93 (10.5%)	234 (12.9%)	327 (12.1%)	
	5: Mildly frail	82 (9.3%)	184 (10.1%)	266 (9.8%)	
	6: Moderately frail	114 (12.9%)	241 (13.3%)	355 (13.1%)	
	7–9: Severely frail	164 (18.6%)	197 (10.8%)	361 (13.4%)	
	Missing	1 (0.1%)	1 (0.1%)	2 (0.1%)	
Charlson	0	90 (10.2%)	207 (11.4%)	297 (11.0%)	0.232
Comorbidity	1	82 (9.3%)	209 (11.5%)	291 (10.8%)	
Index (CCI)	2	112 (12.7%)	227 (12.5%)	339 (12.6%)	
	3	102 (11.5%)	248 (13.6%)	350 (13.0%)	
	4	132 (14.9%)	237 (13.0%)	369 (13.7%)	
	5	127 (14.4%)	222 (12.2%)	349 (12.9%)	
	6	104 (11.8%)	194 (10.7%)	298 (11.0%)	
	7	59 (6.7%)	130 (7.2%)	189 (7.0%)	
	8_over	76 (8.6%)	143 (7.9%)	219 (8.1%)	
BMI	< 18.5	49 (5.5%)	80 (4.4%)	129 (4.8%)	< 0.001
Grouped	18.5–24.9	258 (29.2%)	527 (29.0%)	785 (29.1%)	
(kg/m^2^)	25–29.9	230 (26.0%)	559 (30.8%)	789 (29.2%)	
	≥ 30	206 (23.3%)	528 (29.1%)	734 (27.2%)	
	Missing	141 (16.0%)	123 (6.8%)	264 (9.8%)	
Symptomatic at admission	No	50 (5.7%)	361 (19.9%)	411 (15.2%)	< 0.001
	Yes	834 (94.3%)	1456 (80.1%)	2290 (84.8%)	

*p *value corresponds to the chi square test of independence

The distribution of medical interventions after admission is listed in Table 2. The prevalence of admitted patients requiring oxygen during admission was similar in both waves, but the use of HFNO/CPAP was more prevalent in the second wave whilst invasive ventilation was more prevalent in the first wave. The distribution of patients requiring ICU admission had a similar distribution across waves (263 of 2701, 23.1%). The use of dexamethasone, Remdesivir and Tocilizumab was almost exclusive during the second wave.

**Table 2:** Distribution of medical interventions after admission across waves

Variable/Categories	First wave	Second wave	Total	*p *value
	*N *= 884 (100.0%)	*N* = 1817 (100.0%)	*N* = 2701 (100.0%)	
ICU admission	211 (23.9%)	412 (22.7%)	623 (23.1%)	0.489
Oxygen requirement	668 (75.6%)	1328 (73.1%)	1996 (73.9%)	0.148
Oxygen requirement missing	9 (1.0%)	11 (0.6%)	20 (0.7%)	
HFNO/CPAP	81 (9.2%)	400 (22.2%)	481 (17.8%)	< 0.001
HFNO/CPAP missing	9 (1.0%)	18 (1.0%)	27 (1.0%)	
Invasive ventilation	178 (20.1%)	237 (13.0%)	415 (15.4%)	< 0.001
Invasive ventilation missing	6 (0.7%)	10 (0.6%)	16 (0.6%)	
Dexamethasone	58 (6.6%)	1241 (68.3%)	1299 (48.1%)	< 0.001
Tocilizumab	23 (2.6%)	229 (12.6%)	252 (9.3%)	< 0.001
Remdesivir	0 (0.0%)	575 (31.6%)	575 (21.3%)	< 0.001

*ICU* Intensive Care Unit, *HFNO/CPAP* high flow nasal oxygen/continuous positive airway pressure

*p* value corresponds to the χ^2^ test of independence

### Outcome and follow-up time

A total of 752 patients died over the total time at risk (40,777 person-days); 297 of 884 (33.6%) deaths occurred during the first wave and 455 of 1801 (25.3%) during the second wave. The median time of follow-up for those discharged was 10 days (IQR: 5–22 days) and for those who died was 11 days (IQR: 5–19 days). We found no differences in the overall distribution of the follow-up time across waves. Among those discharged, admissions with lengths of stay (LOS) over 35 days were similar across waves (74 of 587, 11.9% for the first wave and 150 of 1346, 11.1% for the second); LOS between 0 and 7 days were more prevalent in the second wave (562 of 1346, 41.8%) than in the first wave (188 of 587, 32.0%). The median probability of survival was 29 days (95% CI 30–41 days) for the first wave, and 37 days (95% CI 32–47 days) for the second.

### Main results

In this cohort, patients admitted during the second wave of the COVID-19 pandemic, had a (crude) mortality rate 25% lower than that of patients admitted during the first wave (IRR 0.75, 95% CI 0.64, 0.86). Mortality rates, and crude IRR for all variables of interest are shown in Table 1 of the supplementary materials. Overall, mortality rates were 1.19 times higher in men than in women (95% CI 1.03, 1.38) and 1.37 times higher in patients of Asian ethnicity compared to white ethnicity (95% CI 1.12, 1.67). Crude IRR was 1.92 times higher (95% CI 1.27, 3.09) among those aged 60–79 years, and 2.81 times (95% CI 1.85, 4.50) in those aged over 80 years compared to patients younger than 40 years. Mortality increased with increasing levels of frailty (CFS and CCI).

Table 3 shows the crude and adjusted IRR (IRRa) for the effect of wave on mortality on the same set of observations. The strongest confounders of the association in this cohort were dexamethasone, oxygen requirement, symptomatic at admission, CCI, and HFNO/CPAP. Mortality was 43% (95% CI 71%, 52%) lower in the second wave compared to the first wave when adjusting for the effect of dexamethasone; and 16% (95% CI 3%, 27%) lower when adjusting for oxygen requirement. Thus, oxygen requirement is acting as partial positive confounder whereas dexamethasone is acting as a negative confounder in this cohort.

**Table 3:** Crude (IRRc) and adjusted IRR (IRRa) for the effect of wave on mortality

Covariate	*N*	Missing	IRRc	IRRa 95%CI	IRRa	IRRa 95%CI
Dexamethasone	2685	0	0.75	(0.64, 0.86)	0.57	(0.48, 0.69)
Oxygen requirement	2665	20	0.75	(0.65, 0.87)	0.84	(0.73, 0.97)
Symptomatic at admission	2685	0	0.75	(0.64, 0.86)	0.85	(0.73, 0.98)
Charlson Comorbidity Index (CCI)	2685	0	0.75	(0.64, 0.86)	0.70	(0.61, 0.82)
HFNO/CPAP	2658	27	0.73	(0.63, 0.84)	0.69	(0.59, 0.80)
Ventilation	2669	16	0.74	(0.64, 0.86)	0.78	(0.67, 0.90)
Remdesivir	2685	0	0.75	(0.64, 0.86)	0.76	(0.65, 0.89)
ICU admission	2685	0	0.75	(0.64, 0.86)	0.76	(0.66, 0.88)
Tocilizumab	2685	0	0.75	(0.64, 0.86)	0.73	(0.63, 0.85)
Clinical frailty score (CFS)	2683	2	0.75	(0.64, 0.86)	0.76	(0.65, 0.88)
Ethnicity	2685	0	0.75	(0.64, 0.86)	0.74	(0.64, 0.86)
BMI grouped (kg/m^2^)	2421	264	0.88	(0.74, 1.04)	0.89	(0.75, 1.05)
Age grouped (years)	2685	0	0.75	(0.64, 0.86)	0.75	(0.65, 0.87)
Sex	2685	0	0.75	(0.64, 0.86)	0.75	(0.65, 0.87)

Covariates above the dotted line are those change in the magnitude of the effect was ≥ 5.5%

*IRRc* crude RR, *IRRa* adjusted RR, *ICU* Intensive Care Unit, *HFNO/CPAP* high flow nasal oxygen/continuous positive airway pressure

In the multivariable analysis, the hazard of death during the second wave was 1.62 times higher (95% CI 1.26, 2.08) than during the first wave, after conditioning on age, sex, dexamethasone, oxygen requirement, symptoms at admission, and CCI. With age fitted as a flexible spline, and accounting for all the variables in this model, males had HR 1.21 (95% CI 1.04, 1.40); those presenting with symptoms at admission a HR 1.72 (95% CI 1.35, 2.20) and increasing CCI was (non-linearly) associated with increasing hazards of death. Dexamethasone reduced the hazard of death by 53% (95% CI 40%, 63%) when accounting for all the other factors in the model.

In the subgroup analyses of COVID-19 patients requiring ICU, the hazard of death during the second wave was 2.00 (95% CI 1.10, 3.62) after conditioning on age, sex, dexamethasone, Remdesivir, Tocilizumab, and HFNO/CPAP. A summary of model development is presented in the supplementary materials.

## Discussion

This cohort study examined differences in the risk of death of patients requiring in-hospital treatment for COVID-19, during the first and second wave of the COVID-19 pandemic in UK.

The number of COVID-19 admissions was 2.05 times higher in second wave compared to the first wave (1817 vs. 884) and the crude mortality rate was 25% (95%CI 14%, 36%) lower for those admitted during the second wave.

We summarised the distribution of baseline characteristics at admission and medical interventions across waves for the entire study cohort (Table 1). During the second wave, younger admissions with moderate levels of frailty/CCI were more prevalent, compared to either older and/or frailer patients in the first wave. COVID-19 vaccination program (which began in England on 8 December 2020), is likely to have impacted the characteristics of hospitalised patients. National cumulative data up to week ending 28 March 2021 showed an overall vaccine uptake in the population of 42.2% for dose one and 4.7% for dose number two. Uptake among those aged ≥ 80 years was 94.3% for dose one and 40.5% for dose number two; in younger age groups uptake was lower concordant with prioritisation of target groups for vaccination [22, 23]. This could explain some of the observed differences in the distribution of age across waves in our cohort.

In addition to this, during the second wave, we observed an increase of COVID-19-specific treatments as trial data emerged for the use of dexamethasone, Remdesivir and Tocilizumab.

The multivariable analysis attempted to account for all the available factors unequally distributed across waves and also associated with mortality (while avoiding multicollinearity in the model).We found a 1.62-fold increase in the hazard of death (95%CI 1.26, 2.08) in the second wave compared to the first wave, after controlling for the effect of age, sex, dexamethasone, oxygen requirement, symptoms at admission and CCI.

The multivariable model includes two highly correlated variables: dexamethasone therapy and oxygen requirement. These are highly correlated as the benefits of dexamethasone in the management of COVID-19 hospitalised patients have been shown only for patients with hypoxaemia, but not among those with milder disease (without hypoxaemia) [24, 25]. This correlation was only observed in the second wave in accordance with changes in the standard of care as evidence became available. In this cohort, the observed proportion of those who survived among those receiving both oxygen and dexamethasone was similar across waves: 75.5% (37 of 51) during the first wave, and 70.5% (866 of 1228) during the second wave. However, the individual distribution of these two variables was different across waves (Table 2). Therefore, both variables were included in the model, as the level of uncontrolled confounding reduced was larger than the error introduced due to collinear effects. In the final model, dexamethasone reduced the hazard of death in this population of patients by 53%, 95% CI 40%, 63% (after accounting for the effect of age, sex, oxygen requirement, symptoms at admission, CCI and wave).

We further explored the effect of wave on mortality on the subpopulation of patients admitted to ICU, i.e., the most severe COVID-19 patients. All these patients had oxygen therapy so, this variable was not a factor in the main model. Within this sub-group of patients, the hazard of death during the second wave was also larger than in the first wave (HR: 2.00, 95% CI 1.10, 3.62) after accounting for the effect of age, sex, dexamethasone, Remdesivir, Tocilizumab and HFNO/CPAP. This further supports the observation that risk of death in COVID-19 hospitalised patients was higher in the second wave compared to the first wave, when differences in the standard of care and the characteristics of the patients were taken into account.

There is evidence that VOC 202012/01 (alpha) is associated with increased risk of death in the community [12–14]; but since S-gene target failure (SGTF) detection or genomic sequencing data were not available for this study population, attributing our observation of increased in-hospital mortality to variant VOC 202012/01 would largely depend on the acceptability of the assumption that said variant was dominant in our catchment area. This might not be an unreasonable assumption, as community prevalence of SGTF (associated with this new variant), was already at 5.8% at the beginning of November 2020, increasing sharply to reach 94.3% at the end of January 2021 [13]; which correlates with the distribution of second wave admissions (Fig. 1). Supporting this, aggregated St. Georges Hospital data found 84.2% (981 of 1165) of SARS-CoV-2 isolates sequenced between 26 November and 20 February 2021, to be the alpha VOC.

## Strengths and limitations

This was a large analytical cohort study comparing groups of patients at different points in time. The overall goal was to investigate if different standards of care and possible changes in the natural history of the disease (attributed to changes in SARS-CoV-2 variants), had an impact on in-hospital mortality. We included all patients admitted to COVID-19 wards for treatment.

All variables used in this study were extracted prospectively from electronic medical records ensuring data collected were the same across waves. Most of the data were collected by experienced respiratory and ICU clinicians, and although data inconsistencies were verified during data management, misclassification of covariates due transcription errors cannot be ruled out. Additionally, unmeasured variables and/or coarse categorisation (e.g., oxygenation parameters) could have introduced residual confounding.

Overall, there was a good level of data completeness with only BMI observing large numbers of missing values. Data were collected from electronic medical records and a greater number of BMI measurements were not recorded in the first wave as staff were dealing with unprecedented numbers of patients at that time. Across both waves, the proportion of missing values was larger for those with shorter LOS and among those who died. We assessed confounding on complete cases to avoid biasing the estimate.

Outcome and date of outcome were collected separately and ascertained from MCCD (available for 749 of 752 deaths, 99.6%). The number of deaths we observed during the first wave is consistent with numbers previously reported for the same catchment area and period [26]. However, it has been observed that during the first epidemic wave in the UK there was a larger mortality within care homes [2], so it is possible that we have underestimated the number of deaths in the first wave. This differential misclassification of outcome could have led to an overestimation of the effect of the second wave. In addition, temporal effects could also have explained some of the observed differences between waves, as fatality rates are known to be higher during winter months, when the second wave unfolded.

This study was looking at a population of hospitalised adults with COVID-19 in a large reference teaching London hospital. We observed a second wave of admissions twice as large as the first one, which is consistent with national aggregated figures of hospitalisations [3]. The overall demographic characteristics of our cohort (age, sex distribution) are similar to those reported on a large national cohort of patients with COVID-19 admitted to 208 acute care hospitals across England, Wales and Scotland during the growth phase of the first wave [27]. ISARIC WHO CCP-UK reported 17% of ICU admissions and at least, 26% mortality in their cohort (34% patients were in-hospital with no reported outcome, at the time of publication). In our study, we observed larger proportions of both ICU admissions (23.1%, 211 of 884) and deaths (33.6%, 297 of 884) on a comparable period. These differences could be explained in part, by a possible underestimation of deaths among ISARIC admissions with longer LOS, more likely to be sickest (as noted by the authors [27]). In addition, St. Georges Hospital was a regional ICU centre and accepted intubated patients from other hospitals who had run out of ICU capacity, resulting in a population biased towards severity. Our results apply only to an inpatient population, but generalisability will be limited by regional characteristics and factors affecting the force of infection.

## Conclusion

Analysis of COVID-19 admissions recorded in St. Georges Hospital between 01 January 2020 and 31 March 2021, shows a second epidemic wave twice as large as the first one. Although crude rates would indicate a lower in-hospital mortality during the second wave; accounting for differences in the distribution of protective and risk factors (age, sex, dexamethasone use, oxygen requirement, symptoms at admission and comorbidities), suggests a higher risk of death during the second epidemic wave compared to the first. Our findings are temporally and ecologically correlated with an increased circulation of VOC 202012/01 (alpha), with estimates in agreement community-based studies. The availability of improved management and new treatments, particularly dexamethasone, was important in reducing risk of death during the second wave. Our findings highlight the importance of understanding the hospital burden of COVID-19 and the outcomes associated with new circulating SARS-CoV-2 variants.

## Supplementary Information

Below is the link to the electronic supplementary material.

Supplementary file1 (DOCX 51 KB)

## Data Availability

The data underlying this article are available in the article and in its online supplementary material.

## Declarations

### Patient and public involvement

Owing to the nature of this research, no patients or members of the public were involved in the design or reporting of this study. Patient organisations will be involved in the dissemination of these data to inform local management and policy.
